# Phospholipase C β4 promotes RANKL-dependent osteoclastogenesis by interacting with MKK3 and p38 MAPK

**DOI:** 10.1038/s12276-025-01390-8

**Published:** 2025-02-03

**Authors:** Dong-Kyo Lee, Xian Jin, Poo-Reum Choi, Ying Cui, Xiangguo Che, Sihoon Lee, Keun Hur, Hyun-Ju Kim, Je-Yong Choi

**Affiliations:** 1 Department of Biochemistry and Cell Biology, Cell and Matrix Research Institute, School of Medicine, Kyungpook National University, Daegu, Republic of Korea; 2 Department of Internal Medicine and Laboratory of Molecular Endocrinology, Gachon University School of Medicine, Incheon, Republic of Korea

**Keywords:** Bone, Cell signalling

## Abstract

Phospholipase C β (PLCβ) is involved in diverse biological processes, including inflammatory responses and neurogenesis; however, its role in bone cell function is largely unknown. Among the PLCβ isoforms (β1–β4), we found that PLCβ4 was the most highly upregulated during osteoclastogenesis. Here we used global knockout and osteoclast lineage-specific PLCβ4 conditional knockout (*LysM-PLCβ4*^*−/−*^) mice as subjects and demonstrated that PLCβ4 is a crucial regulator of receptor activator of nuclear factor κB ligand (RANKL)-induced osteoclast differentiation. The deletion of PLCβ4, both globally and in the osteoclast lineage, resulted in a significant reduction in osteoclast formation and the downregulation of osteoclast marker genes. Notably, male *LysM-PLCβ4*^*−/−*^ mice presented greater bone mass and fewer osteoclasts in vivo than their wild-type littermates, without altered osteoblast function. Mechanistically, we found that PLCβ4 forms a complex with p38 mitogen-activated protein kinase (MAPK) and MAPK kinase 3 (MKK3) in response to RANKL-induced osteoclast differentiation, thereby modulating p38 activation. An immunofluorescence assay further confirmed the colocalization of PLCβ4 with p38 after RANKL exposure. Moreover, p38 activation rescued impaired osteoclast formation and restored the reduction in p38 phosphorylation caused by *PLCβ4* deficiency. Thus, our findings reveal that PLCβ4 controls osteoclastogenesis via the RANKL-dependent MKK3–p38 MAPK pathway and that PLCβ4 may be a potential therapeutic candidate for bone diseases such as osteoporosis.

## Key role of PLCβ4 in osteoclastogenesis revealed

Osteoclasts are cells that break down bone tissue, and their formation is crucial for bone health. This study explores the role of a protein called PLCβ4 in osteoclast development. Researchers found that PLCβ4 levels increase during osteoclast differentiation, suggesting its importance. The study used mice with specific gene modifications to understand PLCβ4’s role. The researchers used gene knockdown techniques and studied mice lacking PLCβ4 to observe changes in bone cells. They found that reducing PLCβ4 led to fewer osteoclasts and increased bone mass, especially in male mice. This suggests that PLCβ4 is vital for normal bone breakdown. The study concludes that PLCβ4 is crucial for osteoclast development by affecting a signaling pathway involving MKK3 and p38 MAPK. Understanding this process could lead to new treatments for bone diseases such as osteoporosis, where bone breakdown is excessive.

This summary was initially drafted using artificial intelligence, then revised and fact-checked by the author.

## Introduction

Osteoclasts, which originate from monocyte/macrophage lineage cells, are multinucleated cells that are primarily responsible for bone resorption. The differentiation process of osteoclasts from their precursors requires two essential cytokines: macrophage colony-stimulating factor (M-CSF) and receptor activator of nuclear factor κB ligand (RANKL)^1–5^. Osteoclastogenesis is initiated through the interaction of RANKL with its receptor RANK, which leads to the recruitment of the adaptor molecule TNF receptor-associated factor 6 (TRAF6). This RANK–TRAF6 complex triggers the activation of TGF-β-activated kinase-1 (TAK1), which in turn activates the IκB kinase (IKK) complex and mitogen-activated protein kinases (MAPKs), including p38, c-Jun N-terminal protein kinase (JNK) and extracellular signal-regulated kinase (ERK)^6–9^. The activation of these signaling cascades ultimately results in the expression and stimulation of nuclear factor of activated T cells cytoplasmic 1 (NFATc1), a key transcription factor for osteoclast differentiation^10^. During the final stage of osteoclastogenesis, NFATc1 interacts with several transcription factors, including AP-1, PU.1 and microphthalmia transcription factor (MITF), to induce the expression of osteoclast-specific genes, such as tartrate-resistant acid phosphatase (TRAP) and cathepsin K (CTSK)^11^.

Among the three MAPKs, p38 MAPK is a major kinase that plays an essential role in osteoclast differentiation and bone homeostasis. Mice with an osteoclast lineage-specific deletion of p38α generated through the LysM-Cre system exhibit increased bone mass, which is associated with a reduced number of osteoclasts and a reduction in bone resorption^12^. Similarly, mice with Mx-Cre-mediated p38α conditional knockout (KO) exhibit increased bone mass because of the reduced number of osteoclasts and are protected against TNF-mediated inflammatory bone destruction^13^. In addition, specific pharmacological p38 inhibitors have been shown to prevent inflammatory- or ovariectomy-induced bone loss in animal models^14–16^. The expression of the dominant negative form of p38 or treatment with p38 inhibitors completely blocks RANKL-induced osteoclast formation from osteoclast precursor cells in vitro, further supporting these findings^17–19^. Furthermore, p38 MAPK directly stimulates NFATc1 and MITF, thereby inducing the expression of osteoclastogenic markers^20,21^. These studies emphasize the critical role of p38 MAPK activation by RANKL as an essential signaling event required for osteoclast differentiation.

The phospholipase C (PLC) family functions as a critical regulator of phosphoinositide metabolism by hydrolyzing phosphatidylinositol-4,5-bisphosphate into inositol-1,4,5-triphosphate and diacylglycerol^22^. These products function as essential secondary messengers that govern various cellular processes. Dysregulation of PLC function or expression can result in various disorders, such as cancer, neurological disorders and immune dysfunction. The PLC family, which comprises 13 members, is categorized into 6 subfamilies (β, γ, δ, ε, ζ and η) based on their domain structure and mode of activation^23–26^. Within the PLC family, the PLCβ subfamily is further composed of four isozymes (β1, β2, β3 and β4). The expression levels of PLCβ isoforms vary according to tissue type. PLCβ2 is expressed exclusively in hematopoietic cells, whereas PLCβ1 and PLCβ3 are found in various cells and tissues. Meanwhile, PLCβ4 is expressed primarily in neuronal and retinal tissue^27–29^ and has a well-established role in neurological function. Mice that lack PLCβ4 exhibit central nervous system defects such as ataxia, absence seizures and anxiety behavior^30–33^. PLCβ4 deficiency also leads to defects in phototransduction and visual processing^34,35^. In addition, a recent study identified PLCβ4 as a critical regulator of TCR signaling and the immune response^36^, thus highlighting its importance in immune function. Furthermore, mutations in PLCβ4 are associated with a human disease known as auriculocondylar syndrome, also referred to as question-mark ear syndrome, in which patients have developmental defects in their ears and mandible^37,38^. Despite these intriguing findings, the role of PLCβ4 in bone cells, particularly in osteoclasts, remains unknown.

This study revealed that, among the PLCβ isoforms, PLCβ4 presented the highest level of upregulation during osteoclast differentiation. Therefore, we investigated the role of PLCβ4 in RANKL-induced osteoclastogenesis and bone homeostasis by employing a gene knockdown technique and using global KO and osteoclast lineage-specific conditional KO mice for PLCβ4.

## Methods

### Mice

The generation of PLCβ4 global KO mice with a C57BL/6 background has been previously reported^32^. Mice with disrupted PLCβ4 in LysM-expressing cells were generated by crossing LysM-Cre transgenic mice with PLCβ4-floxed mice, and both maintained their C57BL/6 background. All the mice were kept in a specific pathogen-free mouse facility with access to sterilized food, water and bedding under a 12-h/12-h light/dark cycle at 22 °C. All animal procedures were reviewed and approved by the Committee on the Ethics of Animal Experiments of Kyungpook National University (approval no. KNU-2019-0038). The animals were handled in accordance with the relevant guidelines.

### Reagents and antibodies

Recombinant human RANKL was purchased from R&D Systems, and M-CSF was obtained from PeproTech. The PLCβ4-specific antibody was purchased from Santa Cruz Biotechnology. Antibodies against phospho-IκBα, phospho-JNK, phospho-ERK, phospho-p38, phospho-p65, IκBα, JNK, ERK, p38, MKK3 and MKK6 were obtained from Cell Signaling Technology. Antibodies specific for NFATc1 and CTSK were purchased from BD Pharmingen and Millipore, respectively.

### Macrophage isolation and osteoclast culture

Osteoclasts were generated as previously described^39^. In brief, primary bone marrow-derived macrophages (BMMs) were isolated from the long bones of 8–9-week-old mice and cultured in α-minimal essential medium supplemented with 10% fetal bovine serum in a culture dish. The following day, the bone marrow stromal cells and red blood cells were removed, and only the BMMs were collected and cultured in α-minimal essential medium containing 10% fetal bovine serum with 1/10 volume of CMG 14–12 cell culture medium as a source of macrophage colony-stimulating factor^40^ in a Petri dish. After 3 days, the adherent cells were lifted and used as osteoclast precursors (BMMs). BMMs were seeded at a density of 5 × 10^3^ cells per well in a 96-well cell culture plate and cultured with M-CSF (30 ng/ml) and RANKL (20 ng/ml) for 4–5 days to induce osteoclast formation. After the culture period, the osteoclasts were washed with phosphate-buffered saline and fixed with 4% paraformaldehyde for 20 min. Subsequently, the cells were stained with TRAP solution, which consisted of 0.1 M sodium acetate solution (pH 5.0) containing 6.67 mM sodium tartrate, 0.1 mg/ml naphthol AS-MX phosphate and 0.5 mg/ml Fast Red Violet. TRAP-positive multinucleated cells containing three or more nuclei were counted as osteoclasts.

### Microarray analysis

BMMs were cultured for 4 days with 30 ng/ml M-CSF and 20 ng/ml RANKL. The resulting gene expression microarray data were analyzed via Affymetrix gene expression software.

### Lentiviral transduction of PLCβ4 shRNA

The lentiviral constructs containing a PLCβ4 small hairpin RNA (shRNA) or a nonspecific shRNA control (Con-sh) were obtained from Sigma-Aldrich. Lentiviral particles were generated by transfecting 293T cells with the expression constructs, virus packaging plasmid (ΔH8.2) and envelop plasmid (VSVG) via FuGENE HD transfection reagent (Promega). The supernatant containing the constructed lentiviruses was collected after 24–48 h of transfection. BMMs were transduced with the viral mixture along with 10 μg/ml protamine sulfate (Sigma-Aldrich) for 24 h. Subsequently, the cells were subjected to selection by exposure to 4 μg/ml puromycin for 3 days.

### Cell proliferation assay

Cell proliferation was determined via the 3‐(4,5‐dimethylthiazol‐2‐yl)‐5‐(3‐carboxymethoxyphenyl)‐2‐(4‐sulfophenyl)‐2*H*‐tetrazolium (MTS) assay. BMMs were cultured in 96-well plates at a density of 5 × 10^3^ cells per well with various concentrations of M-CSF. After 3 days, the existing medium was removed and replaced with 100 µl of medium containing 20 µl of CellTiter 96 AQueous One solution (Promega). The cells were then incubated for 4 h at 37 °C in a humidified atmosphere with 5% CO_2_, and the absorbance was measured at 490 nm via a microplate reader.

### Quantitative real-time PCR

Total mRNA was isolated from cultured cells, and cDNA was synthesized as previously described^41^. Quantitative real-time polymerase chain reaction (PCR) was performed via an ABI 7500 real-time PCR system with SYBR Green dye (Applied Biosystems). The mRNA expression levels were normalized to those of the endogenous GAPDH housekeeping gene. The sequences of primers used were as follows: PLCβ1, 5′‐CTG CAC AGA GGA TGT GCT GA‐3′ and 5′‐CCA AGT GTC CGA TGT TCC CA‐3′; PLCβ2, 5′‐GCT TCC TCT CCT GTT CAC CC‐3′ and 5′‐CCT TCA CGT TAG GGG GCA AT‐3′; PLCβ3, 5′‐GGA GCG TGT GGA GAG AGC AG‐3′ and 5′‐AGC ACT TCG TTG AGT CTC GG‐3′; PLCβ4, 5′‐TGC CAG ATG GTT TCA CTG AA‐3′ and 5′‐GAA GGT ACC CGC ATG ATC CA‐3′; NFATc1, 5′‐ACC ACC TTT CCG CAA CCA‐3′ and 5′‐TTC CGT TTC CCG TTG CA‐3′; TRAP, 5′‐TCC CCA ATG CCC CAT TC‐3′ and 5′‐CGG TTC TGG CGA TCT CTT TG‐3′; CTSK, 5′‐GGC TGT GGA GGC GGC TAT‐3′ and 5′‐AGA GTC AAT GCC TCC GTT CTG‐3′; OSCAR, 5′-GCT GGC TGC GCT GTG AT-3′ and 5′-ACC TGG CAC CTA CTG TTG CT-3′; Atp6V0d2, 5′‐GAG CTG TAC TTC AAT GTG GAC CAT‐3′ and 5′‐CTG GCT TTG CAT CCT CGA A‐3′; DC‐STAMP, 5′‐CTT CCG TGG GCC AGA AGT T‐3′ and 5′‐AGG CCA GTG CTG ACT AGG ATG A‐3′; and MITF, 5′-GCT ATG CTC ACT CTT AAC TCC AAC-3′ and 5′-TTG GGG ATC AGA GTA CCT AGC TCC-3′.

### Immunoblotting and immunoprecipitation

Cultured cells were washed with ice-cold phosphate-buffered saline and lysed in lysis buffer (50 mM Tris-HCl (pH 7.4), 150 mM NaCl, 1% Nonidet P-40 and 1 mM EDTA) containing protease and phosphatase inhibitors for 10 min on ice. The cell lysates were subsequently centrifuged at 13,000 rpm for 30 min at 4 °C. The protein concentrations of the cell lysates were measured via a Bicinchoninic Acid Kit (Thermo Scientific). Equal amounts of quantified protein from each sample were subjected to 8% or 10% SDS‒PAGE and transferred to polyvinylidene difluoride membranes. After the membranes were blocked with 5% skim milk or 3% bovine serum albumin (BSA), they were incubated overnight with the indicated primary antibodies at 4 °C, followed by probing with the appropriate secondary antibodies. The blots were visualized via an ECL-Plus detection kit (Amersham Pharmacia Biotech). For immunoprecipitation (IP), the cell lysates were incubated with an anti-PLCβ4 antibody or control IgG, followed by Sepharose A beads (GE Healthcare). The immunoprecipitated proteins were separated by 10% SDS‒PAGE and immunoblotted as described above.

### Microcomputed tomography

Mouse femurs were isolated and fixed in 4% paraformaldehyde for 24 h. Subsequently, the femurs were scanned via a Quantum FX μ-CT scanner (PerkinElmer) with a voxel resolution of 9.7 μm, operating at 90 kV and 200 μA. The field of view was set at 10 mm, and the exposure time was 3 min. The region of interest was defined as 0.3 mm from the bottom of the growth plate. Three-dimensional models were constructed from the obtained scan data, and the bone parameters were determined via Analyze 12.0 software.

### Histology and bone histomorphometric analysis

For histological analyses, the proximal tibiae were isolated and fixed in 4% paraformaldehyde for 24 h to evaluate the in vivo osteoclast parameters. Subsequently, decalcification was performed in 10% EDTA for 4 weeks at 4 °C. Following decalcification, the bones were dehydrated, embedded in paraffin and sectioned. The sections were stained with TRAP to visualize the osteoclasts. For dynamic bone histomorphometry analysis, the mice were administered intraperitoneal injections of calcein green (10 mg/kg) and alizarin red (20 mg/kg) on the sixth and second days before being euthanized. The lumbar vertebrae were fixed and embedded in methyl methacrylate resin without decalcification^39^. The resin blocks were cut longitudinally into 6-μm slices of the tibia via a Leica RM2165 rotary microtome equipped with a tungsten blade (Leica Microsystems). The fluorescence signals emitted by calcein green and alizarin red were recorded via a fluorescence microscope (Leica Microsystems). For von Kossa staining analysis, the lumbar vertebrae were fixed and embedded in methyl methacrylate. Subsequently, the 6-μm-thick samples were stained with von Kossa reagent. All the quantitative histological parameters were evaluated via the Bioquant OSTEO II program (BioQuant).

### Immunofluorescence assay

The cells cultured on glass slides in a 24-well plate were fixed with 4% paraformaldehyde for 20 min, permeabilized with 0.1% Triton X-100 and blocked with 0.2% BSA for 10 min. The cells were then incubated for 2 h with primary antibodies in a solution containing 0.2% BSA. To visualize the binding of primary antibodies, fluorescent dye-conjugated secondary antibodies (Molecular Probes) were applied to a 0.2% BSA solution and incubated for 1 h. Immunofluorescence-labeled cells were observed via a fluorescence microscope (Leica Microsystems).

### Statistical analysis

Statistical analyses were performed via Microsoft Excel 2016 (Microsoft) or GraphPad Prism 9 (GraphPad). Statistically significant differences between the two groups were determined via Student’s *t*-test. The data are presented as the mean ± standard deviation (s.d.). A *P* value of <0.05 was considered statistically significant.

## Results

### PLCβ4 expression is upregulated during osteoclastogenesis

**Fig. 1:**
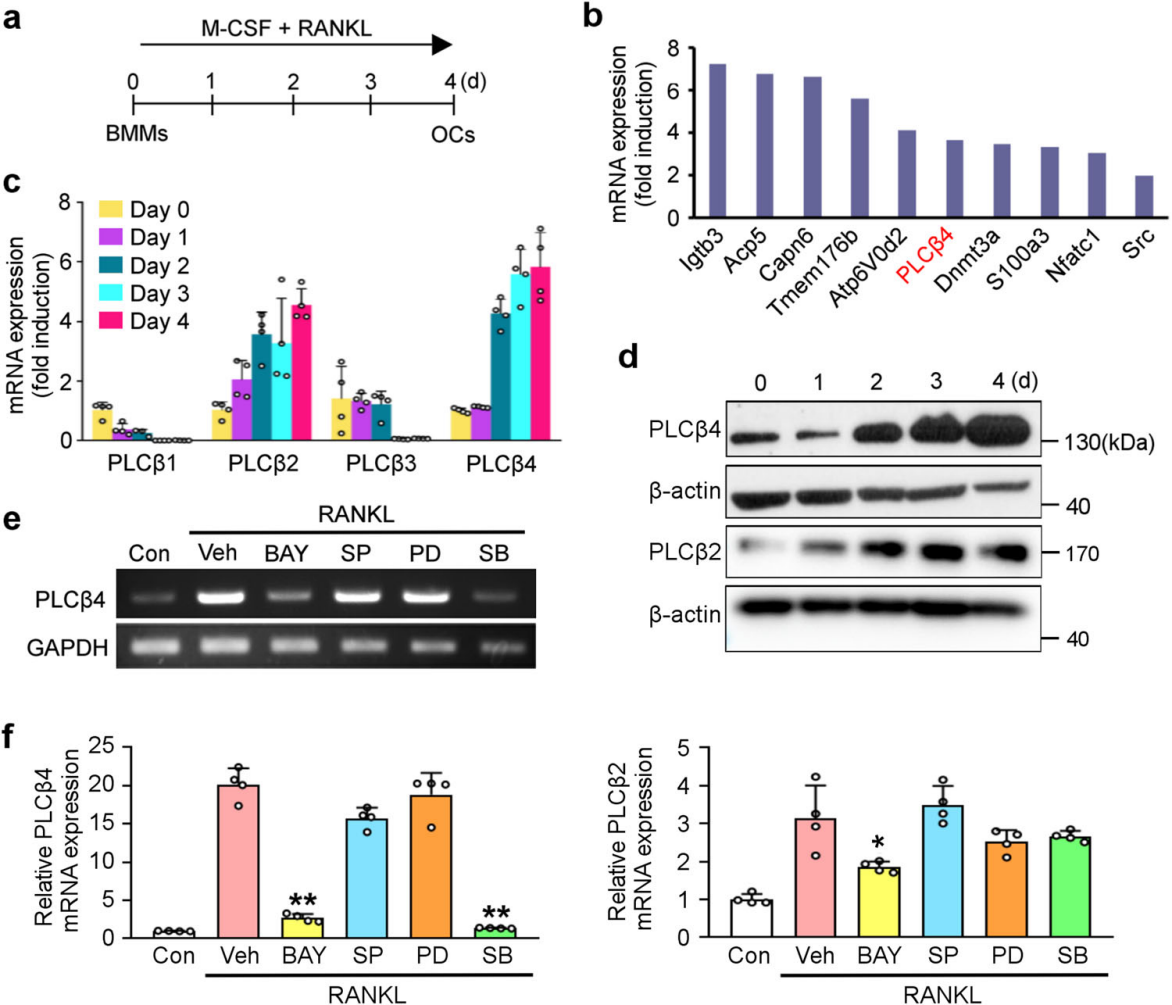
PLCβ4 expression is upregulated during osteoclastogenesis. **a**, BMMs were cultured with 30 ng/ml M-CSF and 20 ng/ml RANKL for 4 days to induce osteoclast (OC) differentiation. **b**, Microarray profile of PLCβ4 expression during osteoclastogenesis. **c**, Quantitative real-time PCR was performed to assess the mRNA expression levels of the PLCβ isoforms (β1–β4) during osteoclastogenesis. **d**, PLCβ4 and PLCβ2 protein levels were determined by immunoblotting. **e**,**f**, BMMs were cultured with M-CSF and RANKL in the presence of the indicated inhibitors. PLCβ4 and PLCβ2 expression levels were analyzed via reverse-transcription PCR (**e**) or real-time PCR (**f**). BAY, Bay11-7802 (2 μM); SP, SP600125 (5 μM); PD, PD98059 (20 μM); SB, SB203580 (10 μM). Data are expressed as mean ± s.d. **P < 0.001 versus the vehicle.

To explore the potential factors that affect osteoclastogenesis, BMMs were cultured with M-CSF and RANKL for 4 days to induce osteoclast generation (Fig. 1a). Next, we performed global transcriptomic analysis using both BMMs and mature osteoclasts. Microarray analysis revealed strong induction of PLCβ4 by RANKL, as well as the upregulation of various osteoclastogenic genes (Fig. 1b). To verify these results, we examined the expression patterns of PLCβ isoforms via real-time PCR. The mRNA levels of PLCβ1 and PLCβ3 decreased during osteoclastogenesis, whereas the mRNA expression of PLCβ4 was most significantly upregulated as the cells differentiated (Fig. 1c). Consistently, the protein level of PLCβ4 was elevated throughout the process of osteoclastogenesis (Fig. 1d). We also observed increased mRNA and protein expression levels of PLCβ2; however, these levels remained lower than those of PLCβ4 (Fig. 1c,d).

We further determined which signaling cascades were involved in the upregulation of PLCβ4 expression by RANKL. To achieve this goal, BMMs were cultured with M-CSF and RANKL in the presence of various inhibitors of the RANKL signaling pathway, such as NF-κB and MAPKs. Our results revealed that the RANKL-mediated increase in PLCβ4 expression occurred mainly via the NF-κB and p38 MAPK pathways, as the increase in PLCβ4 expression was significantly abolished by the NF-κB inhibitor Bay11-7802 and the p38 inhibitor SB203580 (Fig. 1e,f). However, the JNK inhibitor SP600125 and the MEK inhibitor PD98059 did not affect RANKL-mediated PLCβ4 upregulation (Fig. 1e,f). In addition, the RANKL-induced increase in PLCβ2 expression was abrogated by the NF-κB inhibitor (Fig. 1f).

### PLCβ4 knockdown reduces osteoclast differentiation

**Fig. 2:**
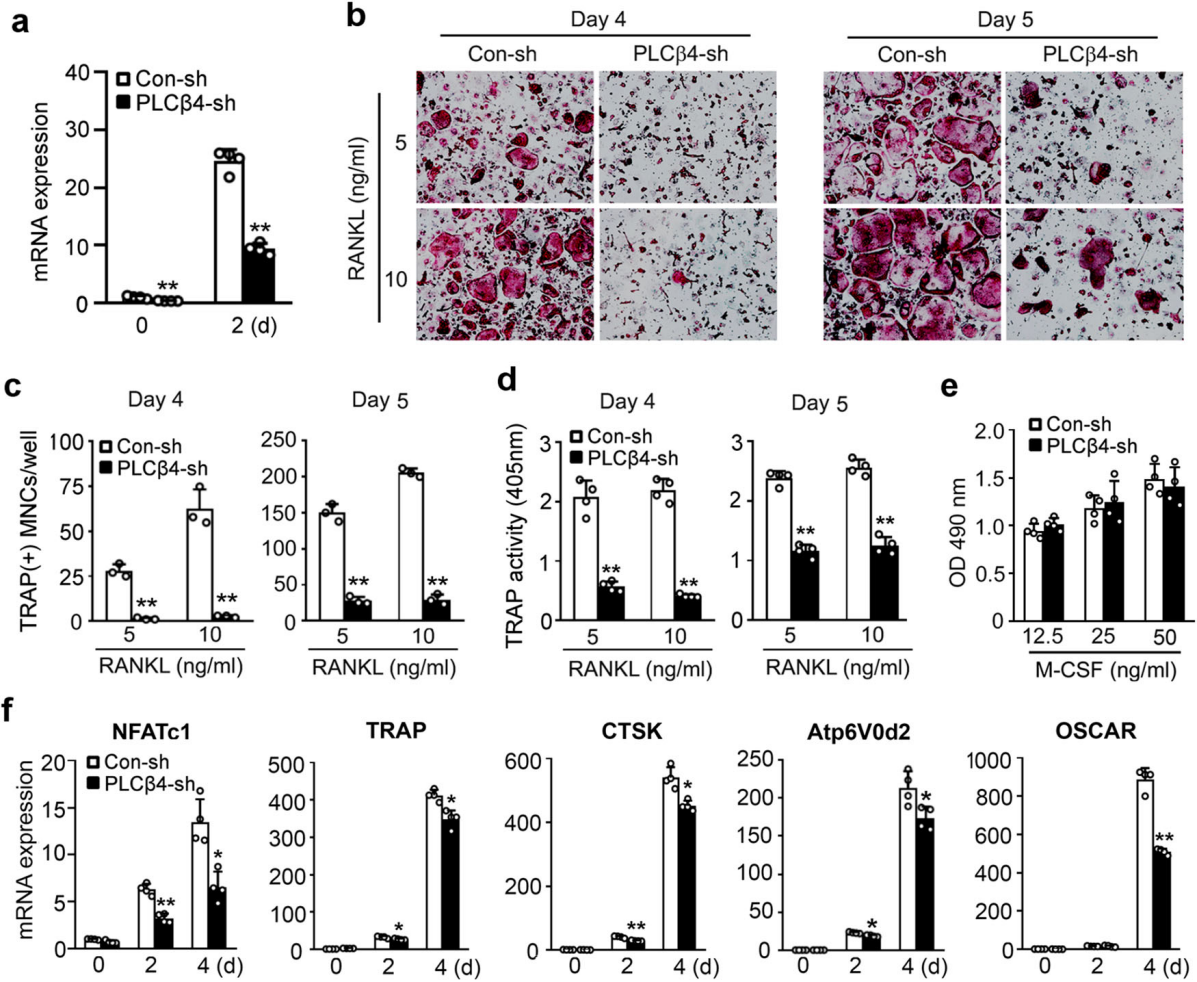
PLCβ4 knockdown reduces RANKL‐mediated osteoclast differentiation. BMMs were transduced with nonspecific control shRNA (Con‐sh) or PLCβ4 shRNA (PLCβ4-sh) lentiviral particles. **a**, The expression of PLCβ4 in BMMs (day 0) or preosteoclasts (day 2) was analyzed by real-time PCR. **b**–**d**, Transduced BMMs were cultured with M-CSF (30 ng/ml) and the indicated doses of RANKL for 4 or 5 days: osteoclasts were visualized after TRAP staining (**b**), and the number of osteoclasts was counted (**c**); TRAP activity (**d**) was determined by using the cultured cell supernatant generated in **b**. **e**, Transduced BMMs were cultured in the presence of various concentrations of M-CSF. After 3 days, the MTS assay was performed as described in the Methods. **f**, Transduced BMMs were cultured in osteoclastogenic medium for the indicated times. Real-time PCR was performed to assess the gene expression of osteoclastogenic markers. All the data are expressed as mean ± s.d. *P < 0.05 and **P < 0.001 versus the control (Con-sh).

The upregulation of PLCβ4 expression via RANKL suggests its potential importance in osteoclasts. To explore this, we used lentivirally mediated expression of a shRNA to silence PLCβ4 expression. As shown in Fig. 2a, lentivirally mediated PLCβ4-shRNA efficiently reduced the mRNA expression of PLCβ4 in BMMs (day 0) and preosteoclasts (day 2). The transduced BMMs were then cultured with M-CSF and two different concentrations of RANKL for 4 or 5 days. The knockdown of PLCβ4 significantly inhibited the formation of TRAP-positive multinucleated cells at both RANKL concentrations (Fig. 2b,c) and decreased TRAP activity in the culture medium (Fig. 2d). However, PLCβ4 knockdown did not affect the proliferation of osteoclast precursors, as determined by an MTS assay (Fig. 2e). We further assessed the impact of PLCβ4 knockdown on the expression of osteoclast-specific markers. PLCβ4 knockdown significantly attenuated the induction of various osteoclastogenic genes, such as NFATc1, TRAP, CTSK, Atp6V0d2 and OSCAR (Fig. 2f).

### Global deletion of *PLCβ4* decreases osteoclastogenesis

**Fig. 3:**
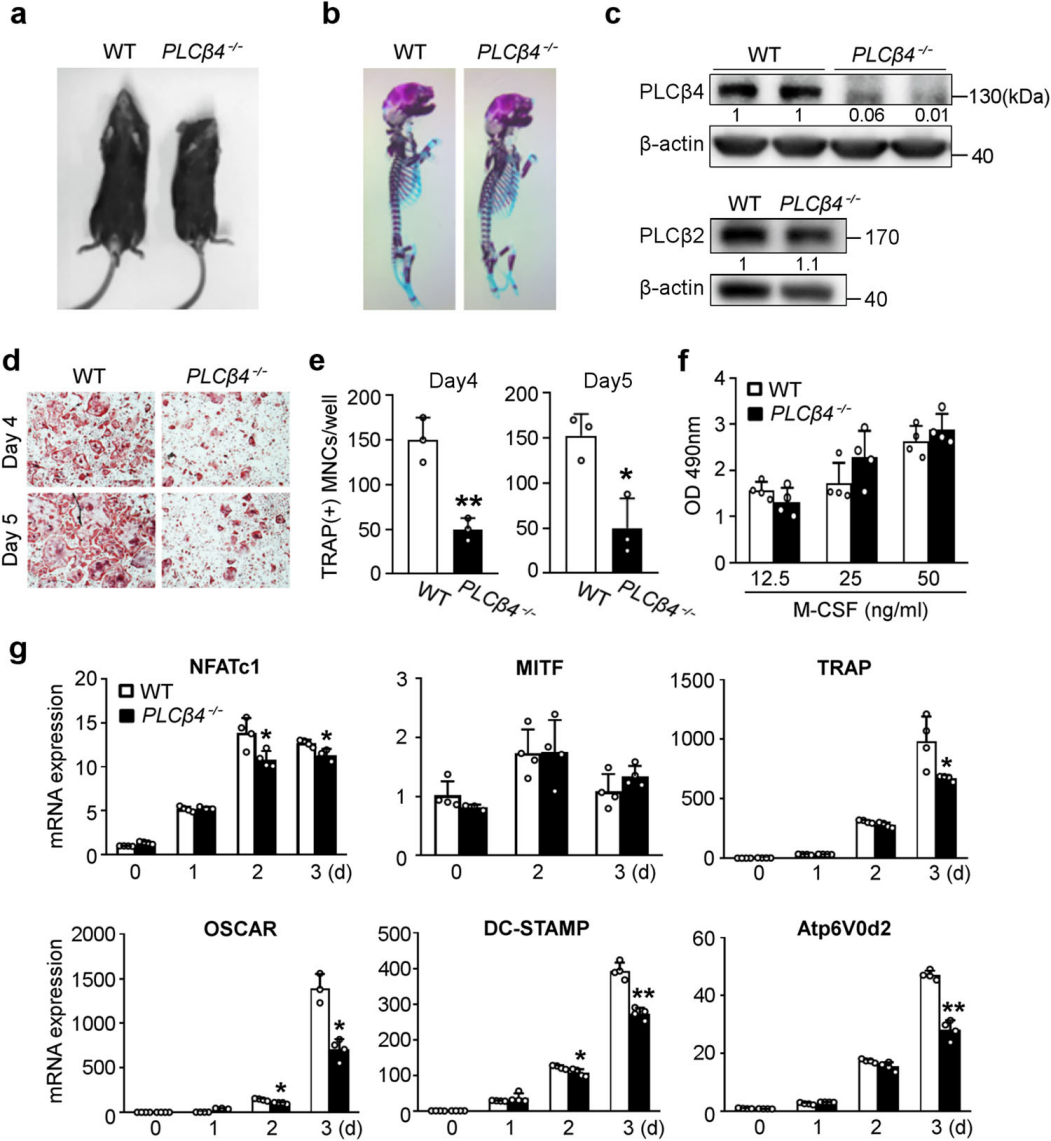
Global deletion of *PLCβ4* reduces osteoclastogenesis. **a**, Size comparison of 8-week-old WT and *PLCβ4*^*−/−*^ mice. **b**, Alcian blue and alizarin red staining of the skeletons of WT and *PLCβ4*^*−/−*^ mice at embryonic day 17.5. **c**, Deletion of PLCβ4 in *PLCβ4*^*−/−*^ BMMs was confirmed by immunoblotting. The relative intensities of PLCβ4 and PLCβ2 are shown after normalization to β-actin. **d**,**e**, BMMs from WT and *PLCβ4*^*−/−*^ mice were cultured with M-CSF (30 ng/ml) and RANKL (20 ng/ml) for 4 or 5 days: the cultured cells were fixed and subsequently subjected to TRAP staining (**d**); the number of osteoclasts was quantified (**e**). **f**, An MTS assay was performed, and the absorbance was measured at 490 nm. **g**, BMMs from WT and *PLCβ4*^*−/−*^ mice were cultured in the presence of M-CSF and RANKL for the indicated days. The expression of osteoclast marker genes was measured by real-time PCR. All the data are expressed as mean ± s.d. *P < 0.05 and **P < 0.001 versus the WT.

Having established that PLCβ4 knockdown reduces osteoclastogenesis, we used PLCβ4 global KO (*PLCβ4*^*−/−*^) mice to further investigate the impact of PLCβ4 deficiency on osteoclasts. Interestingly, the size of 8-week-old *PLCβ4*^*−/−*^ mice was smaller than that of their wild-type (WT) counterparts (Fig. 3a). However, except for mandibular development, no notable differences in skeletal development were observed between the WT and *PLCβ4*^*−/−*^ mice at embryonic day 17.5, as determined by alcian blue and alizarin red staining (Fig. 3b). With respect to osteoclastogenesis, we first confirmed PLCβ4 deletion in *PLCβ4*^*−/−*^ BMMs through immunoblotting analysis (Fig. 3c). Importantly, this deletion did not affect the PLCβ2 protein level, which remained unchanged (Fig. 3c). We subsequently cultured BMMs from WT and *PLCβ4*^*−/−*^ mice in osteoclastogenic media for 4 or 5 days and then performed TRAP staining. The results revealed a significant reduction in the number of osteoclasts in *PLCβ4*^*−/−*^ mice compared with their WT littermates (Fig. 3d,e). Similarly, the gene expression of osteoclast markers, including NFATc1, TRAP, OSCAR, DC-STAMP and Atp6V0d2, was downregulated in *PLCβ4*^*−/−*^ cells compared with WT cells (Fig. 3g). However, there was no significant difference in MITF expression. We further observed that the loss of *PLCβ4* did not influence the proliferation of osteoclast precursors (Fig. 3f).

### Osteoclast lineage-specific deletion of *PLCβ4* reduces osteoclastogenesis

**Fig. 4:**
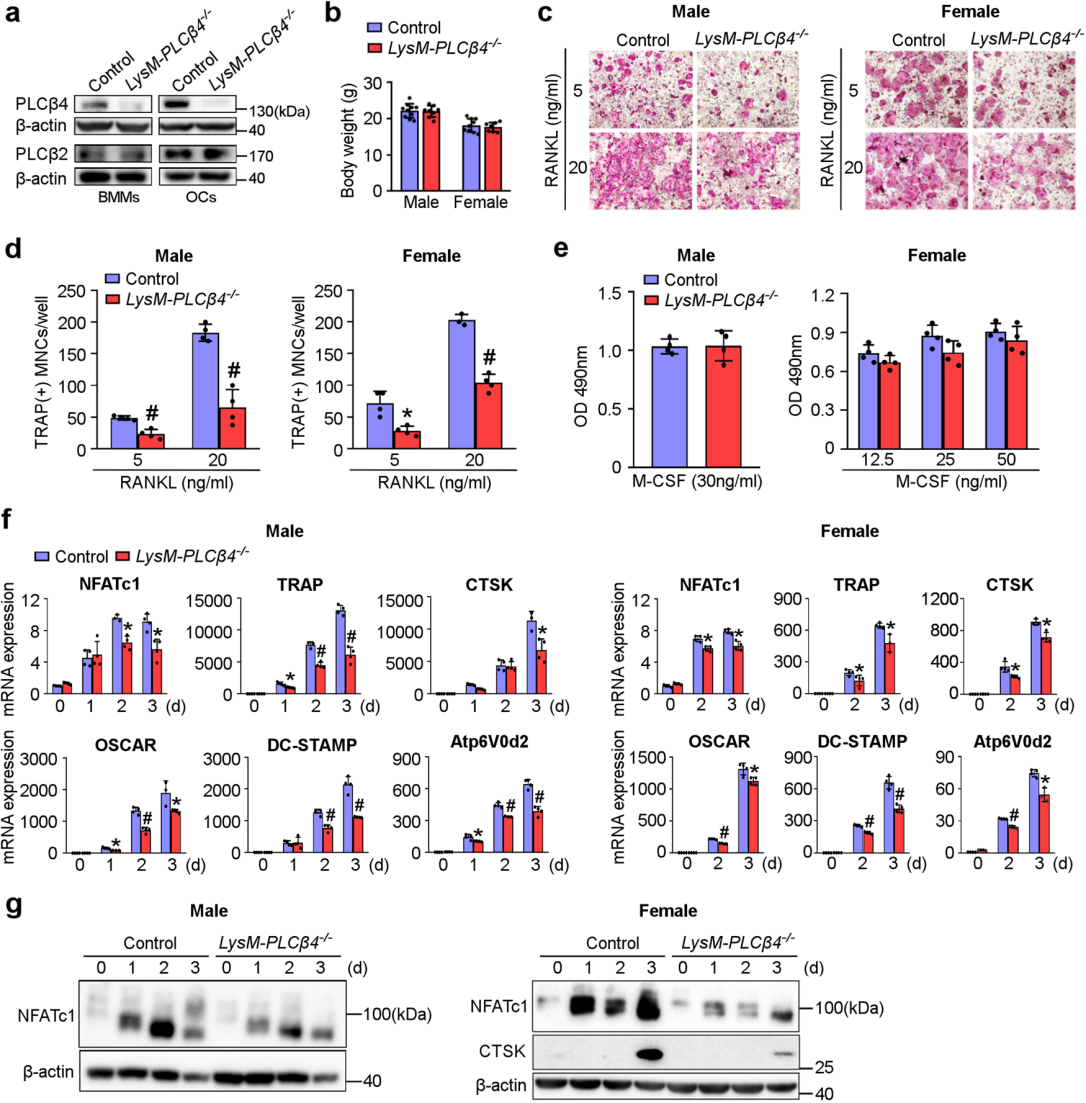
Conditional deletion of *PLCβ4* in the osteoclast lineage reduces osteoclastogenesis. **a**, Deletion of PLCβ4 in *LysM-PLCβ4*^*−/−*^ BMMs and osteoclasts (OCs) was confirmed by immunoblotting. **b**, Comparison of the body weights of 8-week-old control and *LysM-PLCβ4*^*−/−*^ male and female mice (*n* = 7–10). **c**,**d**, BMMs from control and *LysM-PLCβ4*^*−/−*^ male and female mice were cultured with the indicated concentrations of RANKL in the presence of M-CSF (30 ng/ml): osteoclasts were visualized after TRAP staining (**c**): the number of osteoclasts was quantified (**d**). **e**, An MTS assay was performed, and the absorbance was measured at 490 nm. **f**,**g**, BMMs from control and *LysM-PLCβ4*^*−/−*^ male and female mice were cultured in osteoclastogenic medium for the indicated days. The expression of the indicated genes was measured by real-time PCR (**f**) or immunoblotting (**g**). All the data are expressed as mean ± s.d. *P < 0.05 and ^#^*P* < 0.001 versus the control; NS, not significant.

To better understand the involvement of PLCβ4 in osteoclastogenesis, we generated osteoclast lineage-specific PLCβ4 conditional KO mice. To achieve this goal, we crossed *PLCβ4* floxed mice with LysM-Cre mice, which resulted in *LysM-Cre;PLCβ4*^*f/f*^ mice (hereafter referred to as *LysM-PLCβ4*^*−/−*^). For comparison, we used littermates that were homozygous for the *PLCβ4*^*f/f*^ genes but lacked the LysM-Cre allele, and this group was designated as the control. The deletion of PLCβ4 in BMMs and osteoclasts obtained from *LysM-PLCβ4*^*−/−*^ mice was confirmed by immunoblotting (Fig. 4a). Notably, the protein expression of PLCβ2 remained unaltered (Fig. 4a). In addition, neither male nor female *LysM-PLCβ4*^*−/−*^ mice exhibited any substantial gross morphological changes, and their body weights were similar to those of their control littermates (Fig. 4b).

To assess the impact of osteoclast lineage-specific PLCβ4 deficiency on osteoclastogenesis, we cultured BMMs from control and *LysM-PLCβ4*^*−/−*^ male and female mice in the presence of M-CSF and two different concentrations of RANKL. At both concentrations of RANKL, the number of osteoclasts was significantly lower in the *LysM-PLCβ4*^*−/−*^ male and female groups than in the control group (Fig. 4c,d). However, PLCβ4 deficiency did not alter osteoclast precursor proliferation in either the male or female groups (Fig. 4e). In addition, the deletion of PLCβ4 had no effect on the migration or apoptosis of preosteoclasts (Supplementary Fig. 1a,b). Consistent with the reduced formation of osteoclasts, the mRNA expression of various osteoclast-specific marker genes, including NFATc1, TRAP and CTSK, was significantly lower in *LysM-PLCβ4*^*−/−*^ male and female cells than in control cells (Fig. 4f). The reduced expression of osteoclast-specific genes was further supported by the marked reduction in the protein levels of NFATc1 and CTSK in the cells derived from *LysM-PLCβ4*^*−/−*^ male and female mice (Fig. 4g).

### *LysM-PLCβ4*^*−/−*^ male mice exhibit increased bone mass and decreased osteoclast numbers

**Fig. 5:**
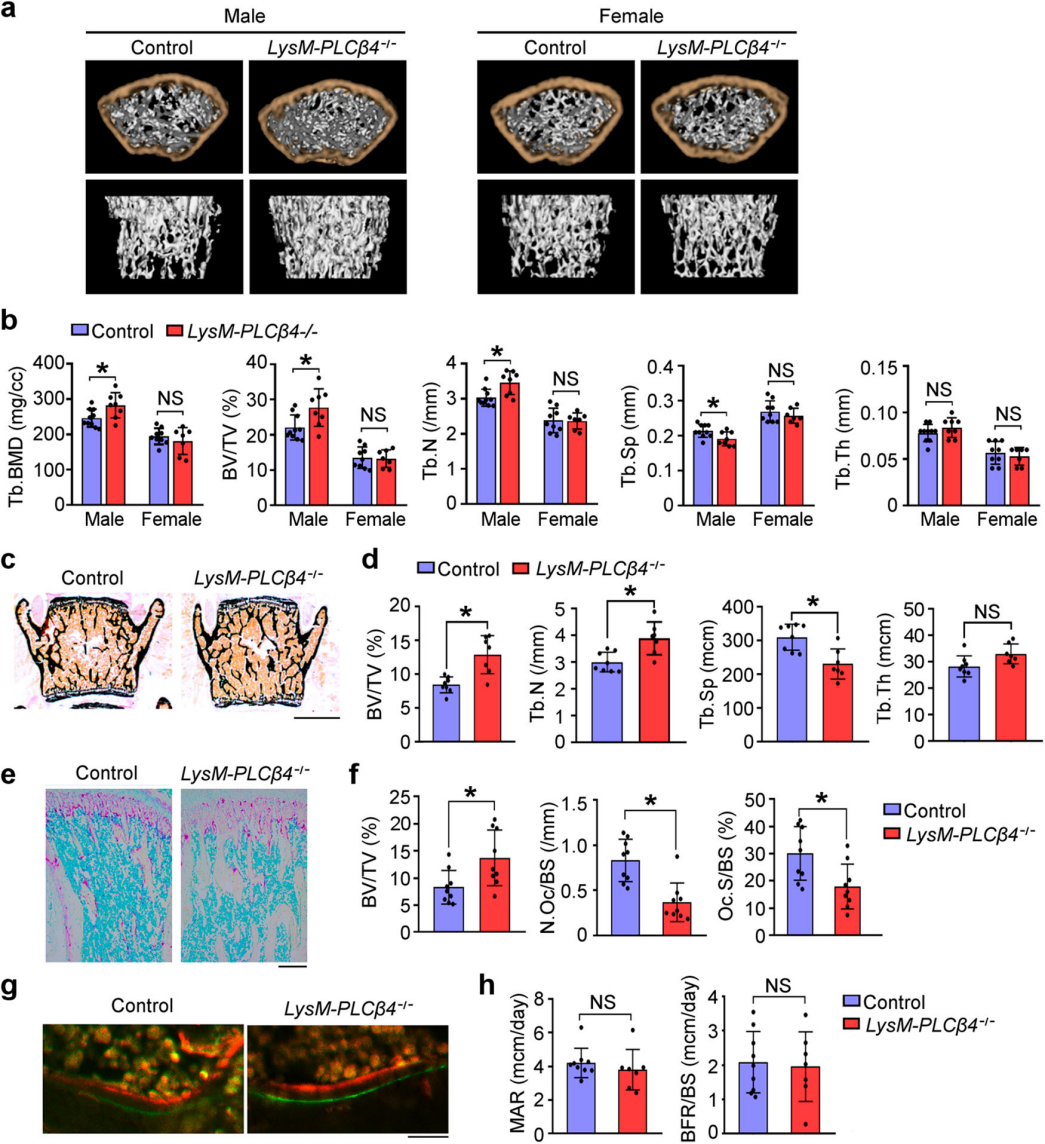
*LysM-PLCβ4*^*−/−*^ male mice have increased bone mass. **a**, Representative µCT images of femurs from 8-week-old control and *LysM-PLCβ4*^*−/−*^ male and female mice. **b**, Quantitative µCT analysis of trabecular bone parameters, including bone mineral density (BMD), bone volume per tissue volume (BV/TV), trabecular number (Tb.N), trabecular separation (Tb.Sp) and trabecular thickness (Tb.Th) (*n* = 7–10). **c**, Von Kossa staining of vertebral sections from 8-week-old control and *LysM-PLCβ4*^*−/−*^ male mice. Scale bar, 1 mm. **d**, Histomorphometric quantification of the data from **c** (*n* = 7–8). **e**, TRAP-stained images of tibias from 8-week-old control and *LysM-PLCβ4*^*−/−*^ mice. Scale bar, 200 μm. **f**, Quantification of the BV/TV, number of osteoclasts per bone surface (N.Oc/BS) and osteoclast surface per bone surface (Oc.S/BS) in **e** (*n* = 9). **g**, Calcein and alizarin red double-labeling images of trabecular bones in vertebrae from 8-week-old control and *LysM-PLCβ4*^*−/−*^ mice. Scale bar, 20 μm. **h**, The mineral apposition rate (MAR) and bone formation rate (BFR/BS) were examined via histomorphometry of the images in **g** (*n* = 7–9). All the data are expressed as mean ± s.d. *P < 0.05; NS, not significant.

To investigate the role of PLCβ4 in bone homeostasis in vivo, we performed three-dimensional microstructural analysis via microcomputed tomography (μCT) on 8-week-old control and *LysM-PLCβ4*^*−/−*^ male and female mice. Interestingly, male *LysM-PLCβ4*^*−/−*^ mice presented significant increases in trabecular bone mineral density, bone volume per tissue volume, and trabecular number. By contrast, no significant changes were observed in the female mice compared with their control littermates (Fig. 5a,b). This increase in bone parameters in male *LysM-PLCβ4*^*−/−*^ mice was accompanied by a reduction in trabecular separation. However, there were no notable differences in the trabecular thickness (Fig. 5a,b). We further found that the osteoclast lineage-specific deletion of PLCβ4 did not affect cortical bone in either sex (Supplementary Fig. 2). Increased bone mass in male *LysM-PLCβ4*^*−/−*^ mice was confirmed by nondecalcified bone histology. Von Kossa staining of lumbar vertebral sections revealed a significant increase in the bone volume per tissue volume and trabecular number and a corresponding decrease in the trabecular separation. By contrast, the trabecular thickness remained unchanged in *LysM-PLCβ4*^*−/−*^ mice (Fig. 5c,d).

To examine whether the increased bone mass in *LysM-PLCβ4*^*−/−*^ mice was due to the reduced number of osteoclasts, we performed histomorphometric analysis on the femurs of 8-week-old *LysM-PLCβ4*^*−/−*^ male mice and their control littermates. Consistent with the μCT data, we detected increased trabecular bone mass in *LysM-PLCβ4*^*−/−*^ mice (Fig. 5e,f). TRAP staining revealed a significant reduction in the number of osteoclasts per bone surface and osteoclast surface per bone surface in *LysM-PLCβ4*^*−/−*^ mice (Fig. 5e,f). However, when the bone formation parameters were examined via calcein and alizarin double labeling, we detected no differences in the mineral apposition rate and bone formation rate between *LysM-PLCβ4*^*−/−*^ mice and their control littermates (Fig. 5g,h). These results indicate that the targeted deletion of *PLCβ4*, specifically in the osteoclast lineage, leads to a decrease in osteoclastogenesis without altering osteoblast formation, which results in greater bone mass in vivo.

### PLCβ4 selectively modulates RANKL-mediated p38 MAPK

**Fig. 6:**
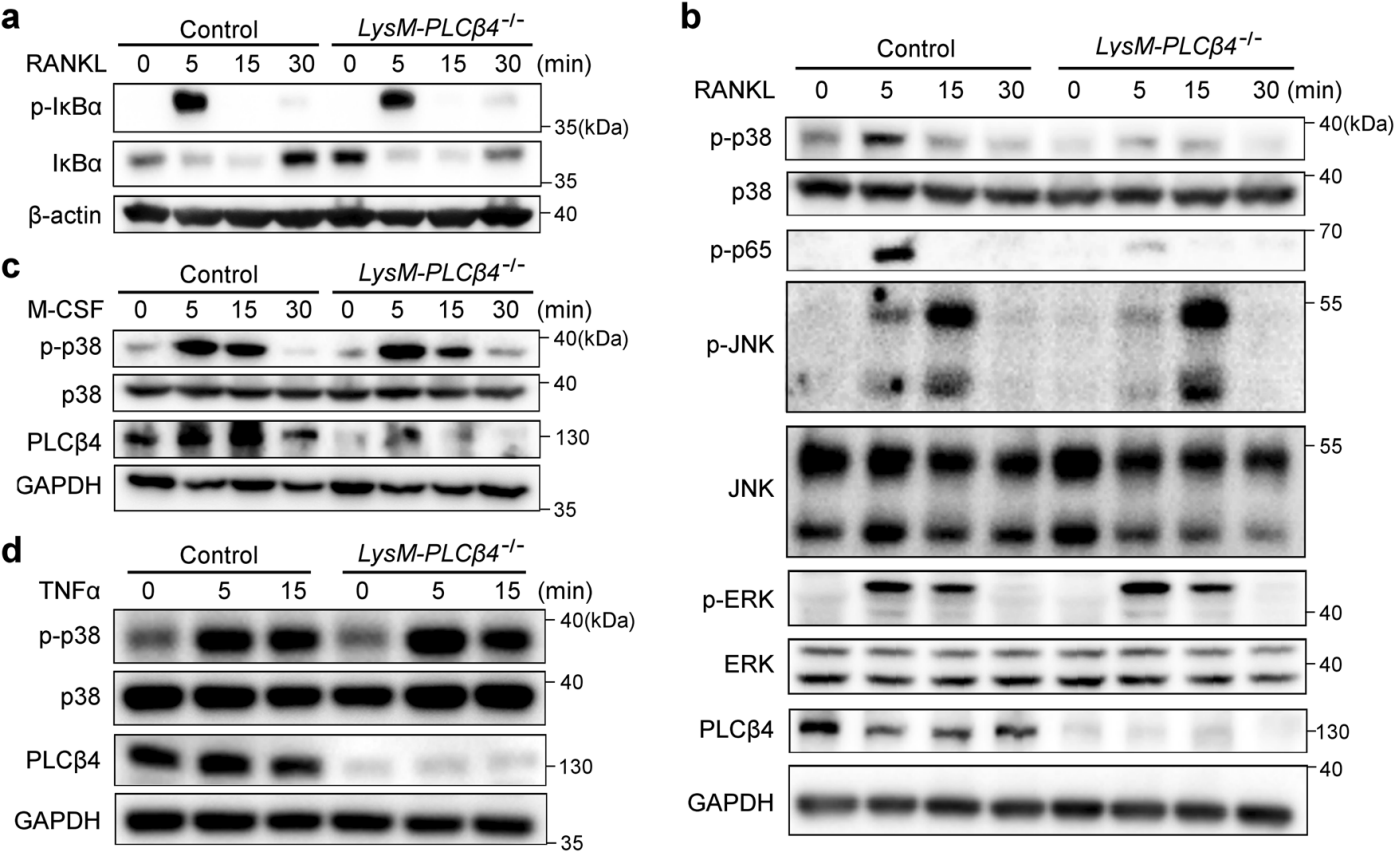
PLCβ4 modulates RANKL-mediated p38 phosphorylation. **a**,**b**, Serum-starved BMMs from control and *LysM-PLCβ4*^*−/−*^ mice were stimulated with RANKL (50 ng/ml) for the indicated times. Immunoblotting was performed to assess IκBα phosphorylation and total IκBα (**a**) and the phosphorylation of p38, p65, JNK and ERK (**b**). **c**,**d**, Serum-starved BMMs from control and *LysM-PLCβ4*^*−/−*^ mice were stimulated with M-CSF (50 ng/ml) (**c**) or TNF (10 ng/ml) (**d**) for the indicated times and analyzed by immunoblotting with antibodies specific for the indicated proteins.

RANKL primarily controls osteoclastogenesis by activating downstream signaling pathways, including NF-κB and MAPKs (JNK, ERK and p38). Therefore, we investigated the molecular mechanism by which PLCβ4 regulates osteoclastogenesis. Serum-starved BMMs from control and *LysM-PLCβ4*^*−/−*^ mice were stimulated with RANKL. The phosphorylation of IκBα, JNK and ERK in response to RANKL stimulation occurred normally in *LysM-PLCβ4*^*−/−*^ BMMs (Fig. 6a,b). However, the activation of p38 was strongly blocked in *LysM-PLCβ4*^*−/−*^ cells compared with control BMMs (Fig. 6b). p38 MAPK mediates RANKL-induced osteoclast differentiation, partially through the phosphorylation of p65 at Ser-536, without affecting IκBα degradation^42^. Thus, we further investigated whether PLCβ4 deficiency influences p65 phosphorylation. We observed that RANKL-stimulated p65 phosphorylation at Ser-536 was markedly reduced in *LysM-PLCβ4*^*−/−*^ BMMs (Fig. 6b). Because M-CSF and TNF also activate the p38 pathway, we next sought to determine whether these cytokines affect p38 signaling in PLCβ4-deficient cells. In contrast to RANKL stimulation, neither M-CSF nor TNF affected p38 phosphorylation (Fig. 6c,d). These results demonstrate that PLCβ4 plays a vital role in osteoclastogenesis by modulating the activation of the p38 signaling pathway in response to RANKL.

### PLCβ4 forms a complex with MKK3 and p38 in response to RANKL

**Fig. 7:**
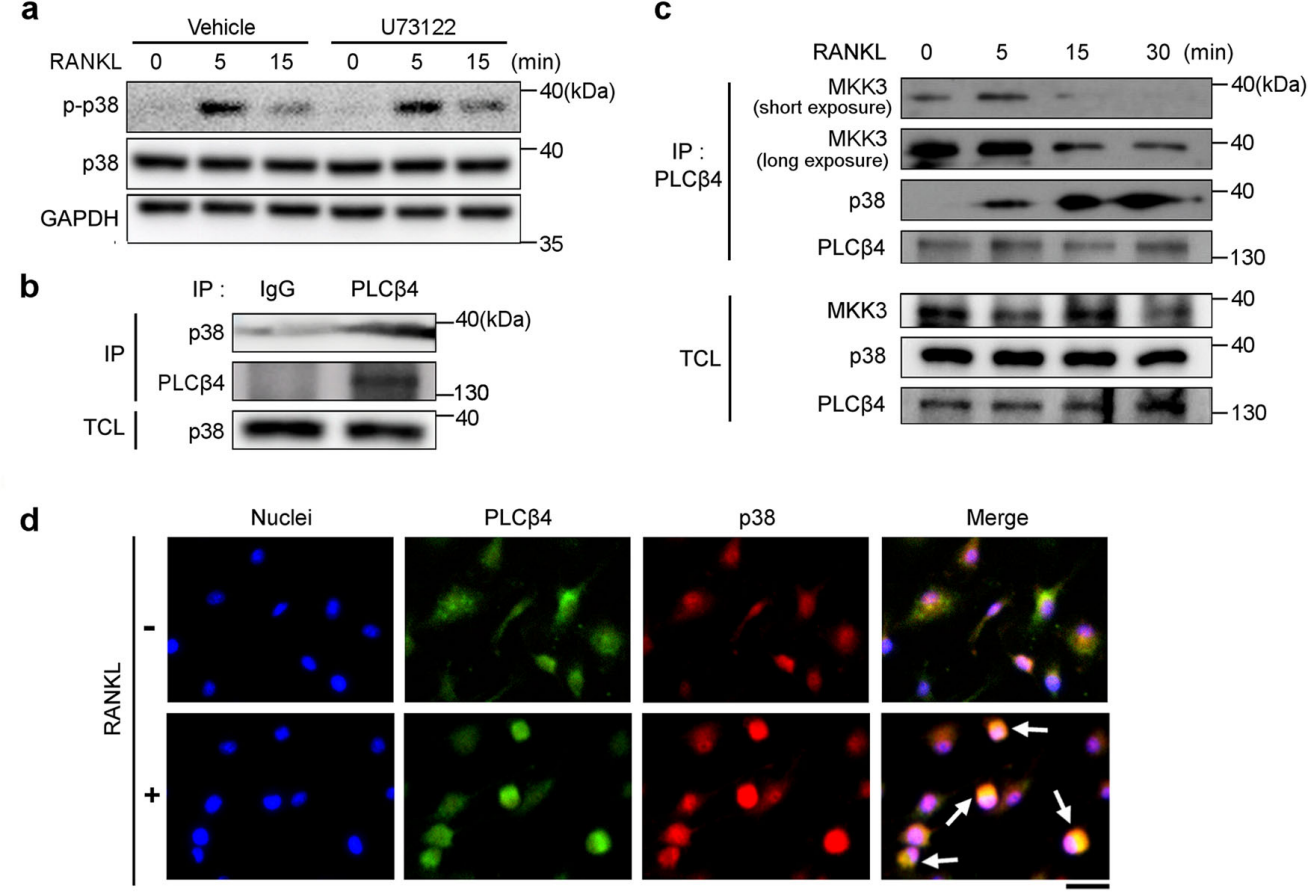
PLCβ4 interacts with p38 MAPK and MKK3. **a**, BMMs from control and *LysM-PLCβ4*^*−/−*^ mice were pretreated with or without the PLC inhibitor U73122 (5 μM) for 1 h and then stimulated with RANKL. Immunoblotting was performed to detect p38 phosphorylation in the cell lysates. **b**, Endogenous PLCβ4 in preosteoclasts was immunoprecipitated and subjected to immunoblotting analysis via anti-p38 and anti-PLCβ4 antibodies. Nonspecific proteins were immunoprecipitated with normal mouse IgG. **c**, Serum- and cytokine-starved BMMs were stimulated with RANKL (50 ng/ml) for the indicated times and subjected to IP with an anti-PLCβ4 antibody, followed by immunoblotting with anti-MKK3, anti-p38 and anti-PLCβ4 antibodies. TCL, total cell lysate. **d**, Serum- and cytokine-starved BMMs were stimulated with or without RANKL (50 ng/ml) for 15 min, fixed and labeled with anti-PLCβ4 antibody (green), anti-p38 antibody (red) and Hoechst dye (blue). The images were detected via a fluorescence microscope. Scale bar, 20 μm.

To investigate whether the catalytic activity of PLCβ4 is essential for RANKL-mediated p38 phosphorylation, control BMMs were pretreated with the PLC inhibitor U73122 and then stimulated with RANKL. As shown in Fig. 7a, the PLC inhibitor did not affect p38 phosphorylation. This observation suggests that the catalytic activity of PLCβ4 is not required for p38 phosphorylation induced by RANKL. Interestingly, PLCβ has been reported to interact with p38 and its upstream regulator MAPK kinase 3 (MKK3), indicating the critical role of PLCβ as an interaction partner with MKK3 and p38 MAPK in cellular signaling processes^43^. Based on these findings and our observation of reduced p38 phosphorylation in PLCβ4-deficient cells, we hypothesized that PLCβ4 can bind to p38 and modulate its phosphorylation. To explore this hypothesis, we conducted an IP assay using preosteoclasts and found that endogenous PLCβ4 directly associates with p38 (Fig. 7b). We then examined the impact of RANKL on the formation of the PLCβ4–MKK3–p38 complex. BMMs treated with RANKL were immunoprecipitated with an anti-PLCβ4 antibody. As shown in Fig. 7c, PLCβ4 was found to interact with MKK3 under basal conditions, and exposure to RANKL further enhanced this interaction, which led to the sequential recruitment of p38 to the PLCβ4–MKK3 complex. To confirm the interaction between PLCβ4 and p38 at the cellular level, we performed a colocalization analysis of these two molecules in BMMs after RANKL stimulation. The results of the immunofluorescence analysis revealed strong colocalization of PLCβ4 and p38 in RANKL-treated BMMs (Fig. 7d).

### Activation of the p38 pathway rescues impaired osteoclastogenesis in PLCβ4 deficiency

**Fig. 8:**
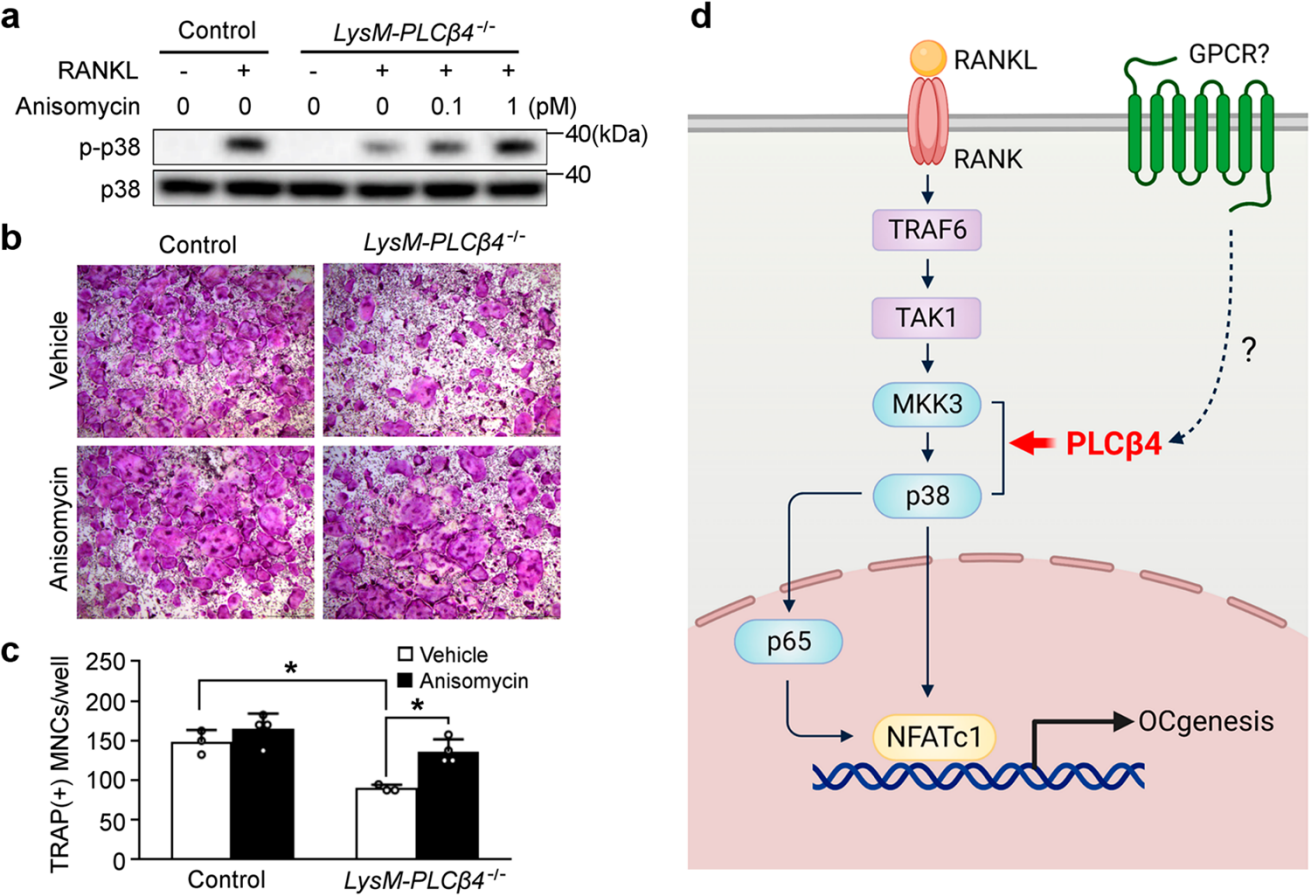
p38 activation restores attenuated osteoclast formation in *LysM-PLCβ4*^*−/−*^ mice. **a**, BMMs from control and *LysM-PLCβ4*^*−/−*^ mice were pretreated with or without the indicated concentrations of the p38 activator anisomycin for 2 h and then stimulated with RANKL (20 ng/ml) for 5 min. Immunoblotting was performed to detect p38 phosphorylation in the cell lysates. **b**,**c**, BMMs from control and *LysM-PLCβ4*^*−/−*^ mice were cultured in osteoclastogenic medium in the presence of anisomycin (1 pM): osteoclasts were visualized after TRAP staining (**b**); the number of osteoclasts was quantified (**c**). The data are expressed as mean ± s.d. *P < 0.05 versus the vehicle. **d**, A schematic diagram of the regulatory mechanism of PLCβ4 in osteoclastogenesis.

Because PLCβ4 deficiency attenuates osteoclastogenesis by blocking p38 phosphorylation, we sought to determine whether activation of the p38 pathway could rescue the impaired osteoclastogenesis observed in *LysM-PLCβ4*^*−/−*^ mice. BMMs from control and *LysM-PLCβ4*^*−/−*^ mice were pretreated with vehicle or the p38 activator anisomycin, followed by RANKL stimulation. The results revealed that the reduced p38 phosphorylation in *LysM-PLCβ4*^*−/−*^ BMMs was restored at two different concentrations of the p38 activator (Fig. 8a). Moreover, treatment with a p38 activator reversed the decrease in osteoclast formation in the absence of PLCβ4 (Fig. 8b,c).

## Discussion

PLC is a key enzyme that maintains cellular homeostasis and normal physiological function. Among the PLC isozymes, PLCγ2 has been extensively studied and is recognized for its critical role in bone metabolism. The absence of PLCγ2 in mice leads to osteopetrosis because of defective osteoclastogenesis^44^. The mechanism underlying this process involves PLCγ2 interacting with the immunoreceptor tyrosine-based activation motif and the adaptor molecule Gab2 to regulate osteoclast differentiation. Although the role of PLCγ2 in bone metabolism is well established, the functions of other PLC isozymes in bone homeostasis remain largely unexplored. In this study, we demonstrated that PLCβ4 plays a crucial role in regulating RANKL-mediated osteoclast differentiation. Our findings reveal that, among the various PLC isozymes, PLCβ4, in addition to PLCγ2, is a critical regulator of bone homeostasis.

Previous studies have demonstrated that PLCβ4 is highly expressed in the brain and retina^27–29^. A recent study reported the detection of PLCβ4 transcripts in CD4^+^ and CD8^+^ splenic T cells, albeit at low levels^36^. Our microarray data revealed higher levels of PLCβ4 expression in mature osteoclasts than in their precursors. These results suggest the potential significance of PLCβ4 in osteoclastogenesis and bone homeostasis. In support of this concept, *LysM-PLCβ4*^*−/−*^ male mice presented increased trabecular bone mass in their femurs and lumbar vertebra. The augmented bone mass observed in PLCβ4 deficiency was accompanied by a reduction in the number of osteoclasts and erosion area on the trabecular bone surface of the femurs, as measured by histomorphometry. Consistent with these in vivo findings, PLCβ4 deficiency led to reduced osteoclastogenesis in vitro in BMMs with PLCβ4 knockdown by shRNA, as well as in BMMs from *PLCβ4*^*−/−*^ and *LysM-PLCβ4*^*−/−*^ mice. Meanwhile, the parameters representing osteoblast activity (mineral apposition rate and bone formation rate) did not significantly change in *LysM-PLCβ4*^*−/−*^ mice. These findings suggest that PLCβ4 deficiency, specifically in osteoclast lineage cells, does not affect in vivo bone formation. Therefore, the increased bone mass observed in *LysM-PLCβ4*^*−/−*^ mice was due to impaired osteoclastogenesis.

A previous study reported that *PLCβ4*^*−/−*^ mice exhibited postnatal lethality due to motor defects^32^. Consistent with our observations, *PLCβ4*^*−/−*^ mice died prematurely at approximately 3 weeks of age. Furthermore, *PLCβ4*^*−/−*^ mice at 8 weeks of age were smaller than WT mice. However, there were no notable differences in skeletal development between the two groups at embryonic day 17.5, except for mandibular development, which indicates that PLCβ4 does not substantially influence bone development during the early stages. In addition, when body weight and size were compared, no difference was detected between the WT and *LysM-PLCβ4*^*−/−*^ mice at 8 weeks of age, which further confirms that the relatively small size of *PLCβ4*^*−/−*^ mice was not due to defects in skeletal development and bone structure.

The sexual dimorphism in bone phenotypes observed in vivo may arise from differences in hormonal regulation, particularly due to the distinct roles of sex hormones in modulating bone metabolism. Notably, we observed sex-related differences in the bone phenotype of *LysM-PLCβ4*^*−/−*^ mice. Such sex-specific skeletal phenotypes are frequently encountered in gene KO animal models, as similar findings have been reported in previous studies^45–49^. Our data revealed that male mice presented alterations in their trabecular bone, whereas female mice did not show any changes. These findings demonstrate a sex-specific response in the absence of PLCβ4, although the precise underlying mechanism remains unclear. Further investigations are needed to fully understand the reasons for the observed sex-specific effects on bone metabolism in the absence of PLCβ4.

Among the MAPKs, p38 plays a crucial role as an essential regulator of NFATc1 induction, which is required for RANKL-mediated osteoclastogenesis, making it a potential target for treating osteoporosis. In this study, we observed that the phosphorylation of p38 in response to RANKL was stunted in the absence of PLCβ4. Notably, PLCβ4 deficiency did not interfere with the activation of p38 MAPK induced by M-CSF or TNF, both of which are pivotal for osteoclast generation. These results demonstrate that PLCβ4 selectively facilitates RANKL-mediated p38 phosphorylation. This regulatory effect of PLCβ4 on p38 activation emphasizes its critical role in mediating RANKL-induced osteoclast differentiation.

Phosphorylation of the NF-κB p65 subunit by RANKL is essential for NF-κB transcriptional activity^42,50^. Notably, treatment with a p38 inhibitor blocks p65 phosphorylation at Ser-536 by RANKL, which subsequently attenuates the transcriptional activity of NF-κB^42^. These observations reveal that p38 MAPK is involved in modulating p65 phosphorylation in response to RANKL. Consistently, we observed a reduction in the phosphorylation of p65 at Ser-536 in *LysM-PLCβ4*^*−/−*^ BMMs. This decrease in p38 and p65 phosphorylation correlated with the attenuated expression of osteoclastogenic markers, such as NFATc1, TRAP and CTSK, in *LysM-PLCβ4*^*−/−*^ cells. Therefore, the reduced phosphorylation of p38 and p65 by RANKL contributes to impaired osteoclastogenesis in the absence of PLCβ4.

The function of p38 MAPK is regulated by two upstream MAPK kinases, MKK3 and MKK6, both of which are known to play essential roles in osteoclast differentiation. In vivo studies using *MKK3*^*−/−*^ and *MKK6*^*−/−*^ mice have shown that both groups exhibit increased bone mass because of impaired osteoclast formation^51^. Interestingly, osteoclastogenesis was significantly decreased in the *MKK3*^*−/−*^ group, whereas MKK6 deficiency had no in vitro impact on osteoclast formation. In addition, the levels of p38 phosphorylation and the expression of osteoclast marker genes, including NFATc1, were significantly attenuated in *MKK3*^*−/−*^ cells but not in *MKK6*^*−/−*^ cells. These findings indicate that MKK3, rather than MKK6, is a principal regulator of osteoclast differentiation in vitro. Our data revealed that PLCβ4 interacts with MKK3 and p38 to form a complex upon RANKL stimulation. In addition, no association between PLCβ4 and MKK6 was observed (data not shown). These data are consistent with those of a previous study using a yeast two-hybrid system and co-IP assays^43^, which supports the observed interaction of PLCβ isozymes with MKK3 and p38 MAPK in this study. Immunofluorescence analysis further confirmed the colocalization of PLCβ4 and p38 following RANKL stimulation. Moreover, treatment with a p38 activator restored defective osteoclast formation in the absence of PLCβ4. Interestingly, the PLCβ4 inhibitor U73122 did not influence p38 phosphorylation, suggesting that the catalytic activity of PLCβ4 is not required for RANKL-mediated osteoclastogenesis. Collectively, these findings highlight the crucial role of PLCβ4 in assembling the components of the MKK3 and p38 MAPK signaling modules to induce p38 activation.

Our study provides convincing evidence that PLCβ4 plays a critical role in maintaining bone homeostasis through the regulation of osteoclastogenesis. We propose a working model whereby PLCβ4, which functions as an adaptor protein, mediates complex formation with MKK3–p38 and the activation of p38 and p65, thus facilitating osteoclast differentiation (Fig. 8d). Our findings emphasize the importance of PLCβ4 in osteoclast biology and suggest its potential as a therapeutic target for managing bone diseases characterized by excessive osteoclast formation.

## Supplementary information

Supplementary Information
