# Supplementary material for: Phospholipase C β4 promotes RANKL-dependent osteoclastogenesis by interacting with MKK3 and p38 MAPK
Source: Exp Mol Med. 2025 Feb 3;57(2):323–34. doi: 10.1038/s12276-025-01390-8 (PMC11873240; doi:10.1038/s12276-025-01390-8)
Supplement: Supplementary file 1 — Supplementary Information [file 12276_2025_1390_MOESM1_ESM.pdf]

Supplementary Figure 1 and legend

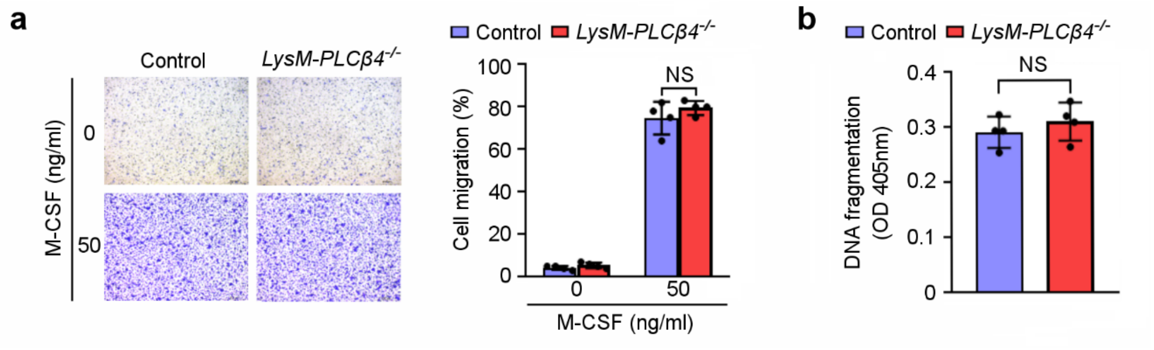

Supplementary Fig. 1 Conditional deletion of *PLCβ4* in the osteoclast lineage does not affect migration or apoptosis of pre-osteoclasts. BMMs from the control and *LysM-PLCβ4*$^{-/-}$ mice were cultured with M-CSF (30 ng/ml) and RANKL (20 ng/ml) for 2 days. **a** Migration assay toward M-CSF (50 ng/ml) was performed. **b** The extent of apoptosis was measured using a DNA fragmentation ELISA.

Supplementary Figure 2 and legend

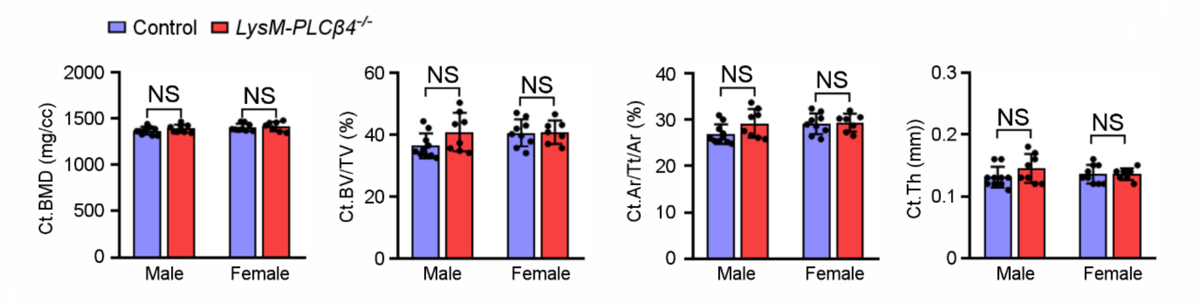

Supplementary Fig. 2 Deletion of *PLCβ4* in the osteoclast lineage does not affect cortical bone parameters. Quantitative μCT analysis of cortical bone parameters of femurs from 8-week-old control and *LysM-PLCβ4*$^{-/-}$ male and female mice. Quantitative measurements of cortical bone mineral density (Ct.BMD), cortical bone volume per tissue volume (Ct.BV/TV), cortical area per total area (Ct.Ar/Tt.Ar), and cortical thickness (Ct.Th) (n = 7–10). NS, not significant
